# Supplementary material for: Implementing allied healthcare professionals in transitional care for older adults with mental health needs: A scoping review
Source: PLoS One. 2026 Mar 30;21(3):e0346032. doi: 10.1371/journal.pone.0346032 (PMC13035120; doi:10.1371/journal.pone.0346032)
Supplement: S1 File — (DOCX) [file pone.0346032.s001.docx]

Ovid MEDLINE(R) ALL

# Query

1 Hospital to Home Transition/ or Patient Transfer/ or Retention in Care/ or Transitional Care/

2 Patient Navigation/

3 (care adj3 (coordinat* or co-ordinat*)).mp.

4 (care adj3 facilitator*).mp.

5 (care adj3 navigator*).mp.

6 (care adj3 transition*).mp.

7 (hospital* adj3 communit*).mp.

8 (hospital* adj3 home*).mp.

9 (navigat* adj3 transition*).mp.

10 (patient* adj3 navigat*).mp.

11 (post-discharge adj3 support*).mp.

12 ((prevent* or reduc*) adj3 (re-admit* or readmit* or re-admiss* or readmiss*)).mp.

13 (preventative* adj3 care).mp.

14 (service* adj3 (coordinat* or co-ordinat*)).mp.

15 (system adj3 navigator*).mp.

16 (transmural adj3 care).mp.

17 or/1-16 [Care transition concept]

18 exp Aged/

19 aging.so.

20 geriatric*.so.

21 elder*.mp.

22 (geriatric* or orthogeriatric*).mp.

23 geriatric*.so.

24 older adult*.mp.

25 ((older adj3 person*) or (older adj3 people)).mp.

26 senior*.mp.

27 or/18-26 [Elderly concept]

28 exp Mental Disorders/

29 Psychological Well-Being/

30 Depression/

31 Obsessive Behavior/

32 exp Substance-Related Disorders/

33 (anxiety or anxious).mp.

34 bipolar.mp.

35 depressi*.mp.

36 hoarding.mp.

37 (obsessive adj3 compulsive).mp.

38 schizophreni*.mp.

39 (addiction* or addictive).mp.

40 (substance* adj3 abuse).mp.

41 substance* use*.mp.

42 or/28-41 [mental health needs concept]

43 and/17,27,42

Embase Classic+Embase, Ovid

# Query

1 hospital to home transition/

2 retention in care/

3 transitional care/

4 (care adj3 (coordinat* or co-ordinat*)).mp.

5 (care adj3 facilitator*).mp.

6 (care adj3 navigator*).mp.

7 (care adj3 transition*).mp.

8 (hospital* adj3 communit*).mp.

9 (hospital* adj3 home*).mp.

10 (navigat* adj3 transition*).mp.

11 (patient* adj3 navigat*).mp.

12 (post-discharge adj3 support*).mp.

13 ((prevent* or reduc*) adj3 (re-admit* or readmit* or re-admiss* or readmiss*)).mp.

14 (preventative* adj3 care).mp.

15 (service* adj3 (coordinat* or co-ordinat*)).mp.

16 (system adj3 navigator*).mp.

17 (transmural adj3 care).mp.

18 or/1-17 [Care transition concept]

19 exp aged/

20 aging.so.

21 geriatric*.so.

22 elder*.mp.

23 (geriatric* or orthogeriatric*).mp.

24 older adult*.mp.

25 ((older adj3 person*) or (older adj3 people)).mp.

26 senior*.mp.

27 or/19-26 [Elderly concept]

28 *mental health/

29 exp *mental disease/

30 *psychological well-being/

31 *depression/

32 *obsession/

33 exp *drug dependence/

34 (anxiety or anxious).mp.

35 bipolar.mp.

36 depressi*.mp.

37 hoarding.mp.

38 (obsessive adj3 compulsive).mp.

39 schizophreni*.mp.

40 (addiction* or addictive).mp.

41 (substance* adj3 abuse).mp.

42 substance* use*.mp.

43 or/28-42 [Mental health needs concept]

44 and/18,27,43

APA PsycInfo, Ovid

# Query

1 Hospital Discharge/

2 Client Transfer/

3 Continuum of Care/

4 (care adj3 (coordinat* or co-ordinat*)).mp.

5 (care adj3 facilitator*).mp.

6 (care adj3 navigator*).mp.

7 (care adj3 transition*).mp.

8 (hospital* adj3 communit*).mp.

9 (hospital* adj3 home*).mp.

10 (navigat* adj3 transition*).mp.

11 (patient* adj3 navigat*).mp.

12 (post-discharge adj3 support*).mp.

13 ((prevent* or reduc*) adj3 (re-admit* or readmit* or re-admiss* or readmiss*)).mp.

14 (preventative* adj3 care).mp.

15 (service* adj3 (coordinat* or co-ordinat*)).mp.

16 (system adj3 navigator*).mp.

17 (transmural adj3 care).mp.

18 or/1-17 [Care transition concept]

19 exp Aged/

20 Elder Care/

21 aging.so.

22 geriatric*.so.

23 elder*.mp.

24 (geriatric* or orthogeriatric*).mp.

25 older adult*.mp.

26 ((older adj3 person*) or (older adj3 people)).mp.

27 senior*.mp.

28 or/19-27 [elderly concept]

29 exp Mental Disorders/

30 Mental Health/

31 Well-Being/

32 Depression (Emotion)/

33 Major Depression/

34 Obsessive Compulsive Disorder/

35 exp "Substance Use Disorder"/

36 (anxiety or anxious).mp.

37 bipolar.mp.

38 depressi*.mp.

39 hoarding.mp.

40 (obsessive adj3 compulsive).mp.

41 schizophreni*.mp.

42 (addiction* or addictive).mp.

43 (substance* adj3 abuse).mp.

44 substance* use*.mp.

45 or/29-44 [mental health needs concept]

46 and/18,28,45

Cumulative Index to Nursing and Allied Health, EBSCO

# Query

1 (MH "Hospital to Home Transition")

2 (MH "Transfer, Discharge")

3 (MH "Transitional Care")

4 (MH "Patient Navigation")

5 TI (care N3 coordinat*) OR AB (care N3 coordinat*) OR SU (care N3 coordinat*)

6 TI (care N3 facilitator*) OR AB (care N3 facilitator*) OR SU (care N3 facilitator*)

7 TI (care N3 navigator*) OR AB (care N3 navigator*) OR SU (care N3 navigator*)

8 TI (care N3 transition*) OR AB (care N3 transition*) OR SU (care N3 transition*)

9 TI (hospital* N3 community*) OR AB (hospital* N3 community*) OR SU (hospital* N3 community*)

10 TI (hospital* N3 home*) OR AB (hospital* N3 home*) OR SU (hospital* N3 home*)

11 TI (navigat* N3 transition*) OR AB (navigat* N3 transition*) OR SU (navigat* N3 transition*)

12 TI (patient* N3 navigat*) OR AB (patient* N3 navigat*) OR SU (patient* N3 navigat*)

13 TI (post-discharge N3 support*) OR AB (post-discharge N3 support*) OR SU (post-discharge N3 support*)

14 TI ((prevent* or reduc*) N3 (re-admit* or readmit* or re-admiss* or readmiss*)) OR AB ((prevent* or reduc*) N3 (re-admit* or readmit* or re-admiss* or readmiss*))OR SU ((prevent* or reduc*) N3 (re-admit* or readmit* or re-admiss* or readmiss*))

15 TI (preventative* N3 care) OR AB (preventative* N3 care) OR SU (preventative* N3 care)

16 TI (service* N3 coordinat*) OR AB (service* N3 coordinat*) OR SU (service* N3 coordinat*)

17 TI (service* N3 co-ordinat*) OR AB (service* N3 co-ordinat*) OR SU (service* N3 co-ordinat*)

18 TI (system N3 navigator*) OR AB (system N3 navigator*) OR SU (system N3 navigator*)

19 TI (transition* N3 care) OR AB (transition* N3 care) OR SU (transition* N3 care)

20 TI (transmural N3 care) OR AB (transmural N3 care) OR SU (transmural N3 care)

21 S1 OR S2 OR S3 OR S4 OR S5 OR S6 OR S7 OR S8 OR S9 OR S10 OR S11 OR S12 OR S13 OR S14 OR S15 OR S16 OR S17 OR S18 OR S19 OR S20

22 (MH "Aged") OR (MH "Aged, 80 and Over") OR (MH "Health Services for Older Persons")

23 SO(aging) OR SO(geriatric*)

24 TI (elder*) OR AB (elder*) OR SU (elder*)

25 TI (geriatric*) OR AB (geriatric*) OR SU (geriatric*)

26 TI (older adult*) OR AB (older adult*) OR SU (older adult*)

27 TI ((older N3 person*) OR (older N3 people)) OR AB ((older N3 person*) OR (older N3 people)) SU ((older N3 person*) OR (older N3 people))

28 TI (senior*) OR AB (senior*) OR SU (senior*)

29 S22 OR S23 OR S24 OR S25 OR S26 OR S27 OR S28

30 (MH "Mental Health")

31 (MH "Mental Disorders+")

32 (MH "Psychological Well-Being")

33 (MH "Depression")

34 (MH "Obsessive Hoarding") OR (MH "Obsessive-Compulsive Disorder")

35 (MH "Substance Use Disorders+")

36 TI (anxiety or anxious) OR AB (anxiety or anxious) OR SU (anxiety or anxious)

37 TI (bipolar) OR AB (bipolar) OR SU (bipolar)

38 TI (depressi*) OR AB (depressi*) OR SU (depressi*)

39 TI (hoarding) OR AB (hoarding) OR SU (hoarding)

40 TI (obsessive N3 compulsive) OR AB (obsessive N3 compulsive) OR SU (obsessive N3 compulsive)

41 TI (schizophreni*) OR AB (schizophreni*) OR SU (schizophreni*)

42 TI (addiction or addictive) OR AB (addiction or addictive) OR SU (addiction or addictive)

43 TI (substance* N3 abuse) OR AB (substance* N3 abuse) OR SU (substance* N3 abuse)

44 TI (substance* N3 use) OR AB (substance* N3 use) OR SU (substance* N3 use)

45 S30 OR S31 OR S32 OR S33 OR S34 OR S35 OR S36 OR S37 OR S38 OR S39 OR S40 OR S41 OR S42 OR S43 OR S44

46 S21 AND S29 AND S45

De-duplicated results, n=8,385

(De-duplicated using Endnote X9)
